# Altered Functional Connectivity and Small-World in Mesial Temporal Lobe Epilepsy

**DOI:** 10.1371/journal.pone.0008525

**Published:** 2010-01-08

**Authors:** Wei Liao, Zhiqiang Zhang, Zhengyong Pan, Dante Mantini, Jurong Ding, Xujun Duan, Cheng Luo, Guangming Lu, Huafu Chen

**Affiliations:** 1 Key Laboratory for NeuroInformation of Ministry of Education, School of Life Science and Technology, University of Electronic Science and Technology of China, Chengdu, People's Republic of China; 2 Department of Medical Imaging, Nanjing Jinling Hospital, Clinical School, Medical College, Nanjing University, Nanjing, People's Republic of China; 3 Institute for Advanced Biomedical Technologies, G. D'Annunzio University Foundation, Chieti, Italy; 4 Department of Clinical Sciences and Bio-imaging, G. D'Annunzio University, Chieti, Italy; 5 Laboratory of Neuro-psychophysiology, K. U. Leuven Medical School, Leuven, Belgium; Cuban Neuroscience Center, Cuba

## Abstract

**Background:**

The functional architecture of the human brain has been extensively described in terms of functional connectivity networks, detected from the low–frequency coherent neuronal fluctuations that can be observed in a resting state condition. Little is known, so far, about the changes in functional connectivity and in the topological properties of functional networks, associated with different brain diseases.

**Methodology/Principal Findings:**

In this study, we investigated alterations related to mesial temporal lobe epilepsy (mTLE), using resting state functional magnetic resonance imaging on 18 mTLE patients and 27 healthy controls. Functional connectivity among 90 cortical and subcortical regions was measured by temporal correlation. The related values were analyzed to construct a set of undirected graphs. Compared to controls, mTLE patients showed significantly increased connectivity within the medial temporal lobes, but also significantly decreased connectivity within the frontal and parietal lobes, and between frontal and parietal lobes. Our findings demonstrated that a large number of areas in the default-mode network of mTLE patients showed a significantly decreased number of connections to other regions. Furthermore, we observed altered small-world properties in patients, along with smaller degree of connectivity, increased n-to-1 connectivity, smaller absolute clustering coefficients and shorter absolute path length.

**Conclusions/Significance:**

We suggest that the mTLE alterations observed in functional connectivity and topological properties may be used to define tentative disease markers.

## Introduction

Human brain function is thought to rely on the two principles of functional specialization and integration. Functional integration is implemented by the complex and reciprocal neural networks in the brain. Brain networks have been depicted in terms of functional connectivity by electroencephalography (EEG) [1], magnetoencephalography (MEG) [2] and functional magnetic resonance imaging (fMRI) [3]–[5], and in terms of structural connectivity by diffusion spectrum imaging (DSI) [6], diffusion tensor imaging (DTI) [7] and morphological studies [8].

Many brain disorders, such as the Alzheimer's disease [9], schizophrenia [10], [11], autism [12], attention deficit/hyperactivity disorder [13] and epilepsy [14]–[17], often present abnormalities in brain networks. The most common type of human medically-intractable epilepsy is mesial temporal lobe epilepsy (mTLE), and its pathologic substrate is usually the hippocampal sclerosis (HS) [18], [19]. It is typically viewed as a network disorder since the bilateral mesial temporal structures, together with a few of cortical and subcortical structures constitute a temporal epileptogenic networks [20]–[24]. During an interictal period, decreased functional connectivity among ipsilateral networks and contralateral compensatory has been reported in a resting state fMRI study [14], and enhanced EEG connectivity in the epileptogenic zone has been found using interictal EEG recordings [25]. Disruptions in functional connectivity within more brain regions related to the interictal epileptic discharges (IEDs) or seizure propagation have been also revealed. For example, hypersynchrony between the thalamus and remote cortical region was found during TLE seizure [26]. Current multi-modality neuroimaging tools have been devoted to map this epilepsy network from various aspects. Several abnormalities in the metabolic, electrophysiological, and structural profiles within the epilepsy network have been already observed [14], [21], [22], [25], [27]–[29].

fMRI studies based on blood oxygen level-dependent (BOLD) mechanism have been increasingly performed to investigate, with high spatial resolution, brain activation related to the epileptogenic networks [30]–[33]. A popular fMRI method to detect brain networks is functional connectivity, based on the temporal correlation between BOLD signals in distant brain regions. Functional connectivity measures in a resting state condition can detect the coherent spontaneous neuronal activity within a brain network [34], [35]. A variety of resting state networks, each showing a definite spatial topography and putatively corresponding to a specific brain function, has been already detected with this approach. Among them, the default-mode network (DMN) is the most famous and important network for the resting condition, as it consistently shows an increased activity during rest than during active and passive cognitive tasks [36]. In healthy subjects, the DMN areas typically comprise the posterior cingulate/precuneus, medial prefrontal cortex, bilateral inferior temporal cortex and bilateral inferior parietal cortex. There is no consensus on the functions of the DMN, although it is often associated with focus on the external environment, or autobiographical memory, envisioning the future, and mind wandering [37]. Disruptions in functional connectivity within the DMN and other networks have been reported in mTLE using different techniques [14], [15], [31], [38]. As an example, Bettus and colleagues have observed a decreased functional connectivity in an epileptogenic network within temporal lobes with a concomitant contralateral compensatory increased connectivity [14]. In addition, our previous studies suggested that the attention network and the perceptual networks were impaired in mTLE [16], [17]. However, such changes in functional connectivity, as well as the global topological properties of the brain networks in mTLE, require further investigation.

In the present study, we aimed at testing the hypothesis that mTLE disease results in an alteration of: 1) the functional connectivity of whole brain network; 2) the n-to-1 connectivity 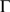, which implicitly describes the amount of information that one region received from the whole network; and 3) the global topological properties of the whole brain functional networks. In this regard, functional connectivity was estimated by calculating the Pearson's correlation between the mean time series of each pair of brain regions for each subject. The resulted correlation matrices were thresholded to generate a set of undirected binary graphs. Therefore, we evaluated topological parameters, the n-to-1 connectivity 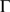, degree of a given node, network hubs, clustering coefficient, shortest path lengths and small-world properties were evaluated.

## Materials and Methods

### Participants

Twenty-three mTLE patients (all right-handed, 8 females, age range: 17-51, mean age 24.1 yrs) participated in the study. We recruited them from May 2005 to October 2008 at the Jinling Hospital, Nanjing University School of Medicine. Some of these patients participated in our previous studies [16], [17]. General information of the patients is summarized in Table S1. All of them underwent a comprehensive clinical evaluation according to the epilepsy classification by the International League Against Epilepsy (ILAE), which included three inclusion criteria: (1) Symptoms of mTLE. Patients had suffered from complex partial seizures; some of them were accompanied by secondarily generalized or simple partial seizures. 11 patients had febrile convulsions in their childhood. (2) MRI manifestation of bilateral hippocampal sclerosis. Hippocampal atrophy [hippocampal volume less than the Chinese normal hippocampus volume (2.62 cm^3^ on the right, and 2.48 cm^3^ on the left <2SDs of the Chinese normal hippocampus volume)] [39], [40] measured in coronal T1 images, and increase in T2 fluid-attenuated inverted recovery (FLAIR) signal in the hippocampus were used as diagnostic criteria. There was no other MRI abnormality than the HS. (3) EEG findings: All patients showed bilateral frontotemporal or temporal lobes interictal discharges on scalp- and sphenoidal EEGs, despite 11 patients were identified as the left sided, and 12 patients as the right sided seizure onset during ictal video-EEG recordings. The exclusive criteria included (1) Structural abnormality other than HS, such as cortical dysplasia, vascular malformation or brain tumor. (2) Unilateral HS or MRI negative in the conventional MRI. Additional details about the patients can be found in our previous studies [16], [17].

Twenty-seven healthy volunteers (all right-handed) were recruited as controls (8 females, mean age, 25.6 yrs). They were recruited among college students and staff components by advertisement at the Nanjing University School of Medicine, and selected to match the patient group in age and gender distribution. They all had no neurological or psychiatric disorder. Written Informed Consents was obtained from all participants. This study was approved by the local Medical Ethics Committee at Jinling Hospital, Clinical School, Medical College, Nanjing University.

### Data Acquisition

MRI data were collected using a 1.5-Tesla scanner (GE-Signa, Milwaukee, US.). Participants were instructed to rest with their eyes closed and to be still. A foam pad was used to minimize the head motion. Firstly, anatomic images were acquired for clinical diagnosis, which included axial T1 weighted images (TR/TE = 2200 ms/24 ms, matrix = 512×512, FOV = 24×24 cm^2^, slice thickness/gap = 4.0 mm/0.5 mm, 23 slices covered the whole brain), coronal T_1_ and T_2_ FLAIR images (4 mm thickness, no gap, 14 slices ) for detecting the hippocampal lesions.

Functional images covering the whole brain were acquired axially using an echo planar imaging sequence (TR = 2000 ms, TE = 40 ms, flip angle = 80°, matrix = 64×64, FOV = 24×24cm; 4mm thickness and 0.5 mm gap, 23 slices). For each subject, the fMRI scanning lasted 7 minutes, thus collecting 210 volumes.

### Data Preprocessing

Data preprocessing was partly carried out using SPM2 (http://www.fil.ion.ucl.ac.uk/spm). The first 10 images were discarded to ensure the magnetization equilibrium. The remaining 200 images were first corrected for the acquisition time delay among different slices, and then were realigned to the first volume for head-motion correction. The time- course of head motion was obtained by estimating the translation in each direction and the rotation in angular motion on each axis for all 200 consecutive volumes. Data of five patients out of 50 subjects were excluded because either translation or rotation exceeded 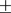
1 mm or 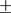
1, respectively. Accordingly, 18 patients (7 females, mean age, 23.9 yrs) and 27 controls (8 females, mean age, 25.6 yrs), matched for age (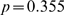, two-sample two-tailed *t*-test) and gender (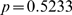, Kruskal-Wallis test), remained for analysis. We also evaluated the group differences in translation and rotation of head motion according to the following formula [11]:
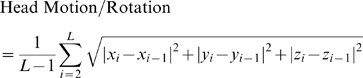
where 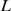 is the length of the time series (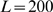 in this study), 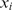, 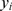 and 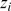 are translations/rotations at the *i*th time point in the 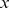, 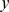 and 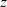 directions, respectively. The results showed that the two groups had no significant differences (two sample two-tailed *t*-test, 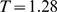, 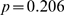 for translational motion and 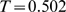, 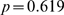 for rotational motion). The fMRI images were further spatially normalized into a standard stereotaxic space at 3×3×3 mm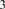, using the Montreal Neurological Institute (MNI) echo-planar imaging template in SPM2 and spatially smoothed by convolution with an isotropic Gaussian kernel (FWHM = 8 mm).

### Anatomical Parcellation

The images were segmented into 90 anatomical regions of interests (ROIs) (45 ROIs for each hemisphere, Table 1) using the anatomically labeled template reported in previous studies [5], [11], [41]. These anatomical ROIs were extracted by the MarsBaR toolbox (http://marsbar.sourceforge.net). For each subject, the representative time series in each ROIs was obtained by simply averaging the fMRI time series across all voxels in the region.

**Table 1:** Summary of network measures for each group.

		Degree (K)				n-to-1 ( 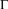 )	
		Controls		mTLE		mTLE vs. Controls	
Region name	Abbreviation	LH	RH	LH	RH	LH	RH
*Medial Temporal*							
Amygdala	AMYG	8	9	10	**11**		
Hippocampus	HIP	5	5	5	5		
Parahippocampal gyrus	PHIP	6	8	7	7		
Middle temporal gyrus, temporal pole	MTGp	2	11	3	3		
Superior temporal gyrus, temporal pole	STGp	10	10	**11**	**13**		
*Subcortical*							
Caudate nucleus	CAU	6	6	4	5		
Olfactory cortex	OLF	8	7	7	7		
Pallidum	PAL	8	8	8	6		
Putamen	PUT	10	11	**11**	**11**		
Thalamus	THA	6	6	5	5		
*Occipital*							
Calcarine fissure	CAL	12	11	**11**	**11**		
Cuneus	CUN	**14**	**14**	**11**	**11**		
Fusiform gyrus	FG	9	**15**	8	**13**		
Lingual gyrus	LING	**13**	**13**	**12**	**13**		
Inferior occipital gyrus	IOG	**14**	**13**	10	10		
Middle occipital gyrus	MOG	**14**	**15**	**13**	10		
Superior occipital gyrus	SOG	**14**	**13**	**13**	**12**		
*Frontal*							
Anterior cingulate gyrus	ACC	**13**	12	8	8		
Inferior frontal gyrus, opercular	IFGoper	**20**	**19**	**13**	**15**		
Inferior frontal gyrus, orbital	IFGorb	**13**	**13**	**11**	**11**		
Inferior frontal gyrus, triangular	IFGtri	**18**	**17**	**11**	10		
Superior frontal gyrus, medial orbital	SFGmorb	**18**	**19**	**20**	**15**	*	
Middle frontal gyrus, orbital	MFGorb	12	**13**	7	8		*
Middle frontal gyrus	MFG	**20**	**19**	**14**	**11**		
Superior frontal gyrus, medial	SFGmed	**19**	**20**	**14**	**22**		*
Superior frontal gyrus, orbital	SFGorb	8	11	10	**11**		
Superior frontal gyrus	SFG	10	**20**	**11**	**12**		
Gyrus rectus	REG	11	12	10	10	*	*
*Temporal*							
Heschl gyrus	HES	11	9	7	7		
Insula	INS	**19**	**18**	**16**	**16**		
Inferior temporal gyrus	ITG	**16**	**24**	7	**13**		
Middle temporal gyrus	MTG	**13**	**19**	**11**	**14**	*	
Superior temporal gyrus	STG	**15**	**15**	**11**	**12**		
*Parietal-(pre)Motor*							
Rolandic operculum	ROL	**14**	**14**	**11**	**13**		
Angular gyrus	ANG	**16**	**18**	**14**	**12**		
Median cingulate gyrus	MCC	**13**	**15**	8	10		
Posterior cingulate gyrus	PCC	**14**	12	10	8		
Paracentral lobule	PCL	4	6	3	6		
Inferior parietal gyrus	IPG	**19**	**17**	**18**	**12**		
Superior parietal gyrus	SPG	9	11	6	4		
Postcentral gyrus	PoCG	**14**	12	8	9		
Precentral gyrus	PreCG	**17**	12	**12**	6		
Precuneus	PCUN	**17**	**15**	**11**	**12**		
Supplementary motor area	SMA	**13**	10	**11**	10		
Supramarginal gyrus	SMG	**17**	**19**	**12**	**12**		

The abbreviations listed are those used in this paper, which differ slightly from the original abbreviations by Tzourio-Mazoyer et al. [41]. Six main groups derived from Salvador et al. [5]. Network hubs defined as a node with degree larger than the average degree of the network for each group were listed in bold. An asterisk (*) indicates that the significant stronger n-to-1 connectivity 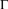 in the patients than the healthy controls. Separate columns show data for left to right cerebral hemispheres (LH and RH, respectively).

### Functional Connectivity and Graph-Theory Preprocessing

Several procedures were used to remove the possible spurious variances from each regional (ROI) mean time series [5], [11]: 1) Temporal band-pass filtering (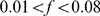
Hz), which was performed in order to reduce the effects of low-frequency drift and high-frequency noise [11], [34], [35]. 2) Each time series was further corrected for the effect of head motion parameters [5], [13], [14] by linear regression. 3) Each time series was also corrected for the ventricular signal averaged from a ventricular ROI and 4) the white matter signal averaged from a white matter ROI through linear regression according to previous resting state fMRI studies [14], [34], [42]. 5) The residuals of these regressions were linearly detrended [13], and then used for the functional connectivity and graph-theory analysis.

### Computation of Correlation Matrix

The resting state BOLD time series were correlated region by region for each subject across the full length of the resting time series (L = 200 time points, d.f. = 197), and then a square 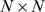 (where 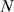 = 90 is the number of ROIs) correlation matrix was obtained for each subject. A Fisher's r-to-z transformation was applied to the correlation matrices to improve the normality of the correlation coefficients (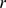) [11]. For each group, z-score matrices were averaged across all subjects in each group [5], [11].

### Graph Visualization

The regional centroid of each ROI (node) was positioned according to its anatomical location in the MNI stereotaxic space by using Pajek software [43] (http://vlado.fmf.uni-lj.si/pub/networks/pajek/). The edges (functional connectivity) between nodes could be constructed by applying a correlation threshold 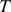 (Fisher's r-to-z). We defined the threshold in terms of probability that the observed 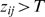 under the null hypothesis that 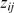 is less than an arbitrary value 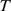. As the possible 4005 (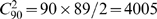) inter-regional correlations were subjected to multiple, non-independent tests, we employed the strict Bonferroni correction for multiple comparisons (i.e., 0.001/4005 = 2.4969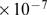 as threshold).

### Direct Comparisons between Patients vs. Controls

We performed two-sample two-tailed *t*-test on all 4005 possible connections represented in the two 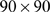 correlation matrices related to patients and controls [11], [42]. To account for multiple comparisons, the false discovery rate (FDR) method was applied [42].

### Graph-Theory Analysis

#### Topological properties of the brain functional networks

The topological properties of the brain functional networks were defined on the basis of a 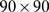 binary graph, 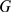, consisting of nodes and undirected edges (see *Graph visualization*):
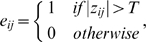
where 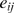 refers to the edge in the graph. In general, if the absolute 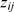 of a pair of brain regions, 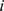 and 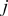, exceeds a given threshold 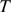, an edge is assumed to exist; it does not exist otherwise. A subgraph 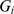 is defined as the graph including the nodes that are the direct neighbours of the *i*th node, i.e. directly connected to the *i*th node with an edge. The degree at each node, 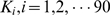, is defined as the number of nodes in the subgraph 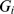. The degree of connectivity of a graph, 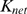, is the average of the degrees of all the nodes in the graph:
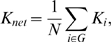
which is a measure for the sparsity of a network. Briefly, the degree of a given node, 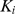, denotes to which extent the node is connected to the rest of the network. A node with a higher degree has more connections (where each connection is counted once) [6].

#### Network hubs

After creating the brain network using the selected threshold, we then determined which nodes were connected to the largest number of other nodes, i.e. which nodes are “hubs” [1], [6]. Specifically, we define a hub as a node whose degree is larger than the average degree of the network [2].

#### Degree distribution fits

More details about the degree distribution can be found in Text S1 and Table S2.

#### n-to-1 Connectivity 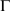


Based on the studies of Jiang et al. [44], the connectivity degree 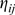 between the node 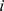 and the node 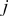 was expressed as 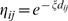 [44], [45]. 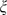 is a real positive constant, which measures how the strength of the relationship decreases along with the distance between two nodes. It was set to 2 in this study [44], [45]. 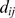 refers to the distance between the two nodes, and was calculated as: 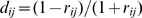, where 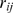 represents the correlation between two brain regions 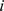 and 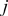. Therefore, the total connectivity degree 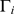 of a node 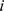 in a graph is the sum of all the connectivity degrees between node 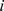 and all other nodes, i.e., 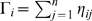 [44]. It describes the amount of information that the node 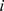 receives from the particular network. Obviously, 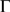 differs from the canonical cross-correlation analysis using the 1-to-1 connectivity measures delineated above. It may be possible to find changes of the total functional connectivity degree in different brain activity states [44]. We further normalized 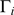 of a node 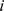, namely, 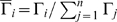. The differences of 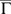 of each ROI between mTLE patients and healthy controls were tested using a two-sample two-tailed *t*-test, with FDR correction.

#### Clustering coefficient

The absolute clustering coefficient of a node is the ratio between the number of existing connections and the number of all possible connections in the subgraph 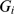:
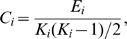
where 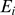 is the number of edges in the subgraph 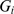 [46], [47]. The absolute clustering coefficient of a network is the average of the absolute clustering coefficient of all nodes:
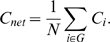


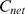
 is a measure of the extent of the local density or cliquishness of the network.

#### Shortest path lengths

The mean shortest absolute path length of a node is:
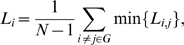
in which 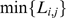 is the shortest absolute path length between the node 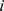 and 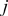, and the absolute path length is the number of edges included in the path connecting two nodes. The mean shortest absolute path length of a network is the average of the shortest absolute path lengths between the two nodes:
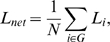


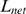
 is a measure of the average connectivity extent, or overall routing efficiency, of the network.

### Small-World Brain Networks

Compared to random networks, which are characterized by a low clustering coefficient and a typical short path length, small-world networks have similar absolute path length but higher absolute clustering coefficient, that is 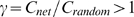, 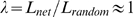 [46]. Those two conditions can also be summarized into a scalar quantitative measurement, namely small-world-ness, 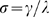, which is typically 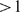 for networks with a small-world organization [3], [48]. To examine the small-world properties, the value of 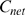 and 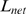 of the functional brain network need to be compared with those of random network (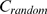 and 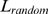).

#### Generation of the random network

The theoretical values of these two measures for a random network are 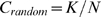, and 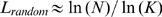 [3], [49], [50]. As suggested by Stam et al. [50], statistical comparisons should generally be performed between networks that have equal (or last similar) degree sequence; however, theoretical random networks have Gaussian degree distributions that may differ from the degree distribution of the brain networks. According to a previous study [11], to obtain a better control for the functional brain networks, we generated 100 random networks for each 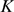 and 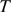 of each individual network by a Markov-chain algorithm [51], [52]. In the original matrix, if node 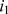 was connected to node 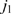 and node 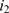 was connected to node 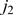 for random matrices, the edge between node 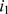 and node 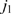 was removed but an edge between node 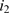 and node 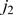 was added. That means that a pair of vertices 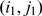 and 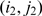 was selected for which, 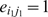, 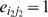, 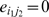, and 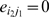. Then 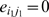, 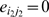, 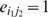 and 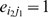. Then we randomly permuted the matrix which assured that random matrix had the same degree distribution as the original matrix. We repeated this procedure until the topological structure of the original matrix was randomized [3]. Then we averaged across all 100 generated random networks to obtain a mean 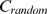 and a mean 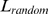 for each degree 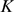 and threshold 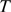.

#### Identifying small-world regime

Although there is currently no formal consensus regarding threshold selection, we investigated the topological properties of brain functional network as a function of 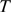 and 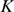, following the studies by Stam and colleagues [50] and Liu and colleagues [11]. (1) We thresholded all matrices using a single, conservative threshold chosen to construct a sparse graph with mean degree 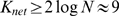 (total number of edges 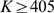). The maximum threshold 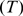 is selected also to assure that each network is fully connected with 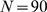 nodes. This allowed us to compare the topological properties between the two groups in a way that was relatively independent of the size of the network. (2) The minimum threshold is selected to ensure that the brain networks have a lower global efficiency and a larger local efficiency compared to random networks with relatively the same distribution of the degree of connectivity [4]. We selected the threshold range, 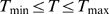 by intersecting the upper criteria. As shown in Figure S1, we selected the small-world regime as 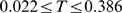 (with steps of 0.005), which corresponded to the degree of connectivity threshold 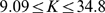 (with steps of 0.83).

### Correlation between Topological Measures and Clinical Variables

To investigate the underlying relationship between properties measures (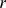, 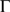, 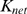, 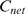, 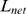, 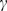, 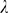 and 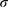) of the brain functional networks and clinical variables (epilepsy duration, seizure frequencies) for each 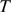 and 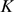 in the mTLE patients group, the Pearson's correlation analysis was used. As these analyses were exploratory in nature, we used a statistical significance level of 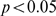, uncorrected.

## Results

### Functional Connectivity of Patients and Healthy Controls

The mean correlation matrix was calculated by averaging the correlation matrix (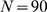 ROIs) across all the subjects within groups (including both positive and negative values). These 90 regions were categorized into six main locations (Table 1 shows the abbreviation corresponding to each ROI) as proposed by Salvador et al. [5]. For better visualization of the structural patterns within those connection matrices, a layout of nodes (individual ROIs) and undirected edges (functional connectivity) were represented as networks (Figure S2).

### Direct Comparisons between Patients and Controls

**Figure 1:**
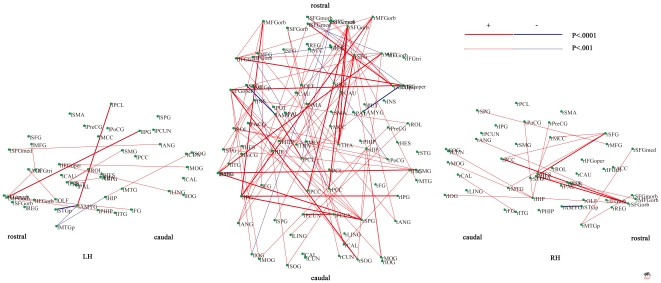
Statistically significant differences in functional connectivity between patients and controls. Nodes (individual ROIs) were differently colored according to the six anatomical subregions listed in Table 1 (see legend). Undirected edges were differently colored according to the significantly larger functional connectivity (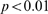 and 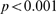, FDR corrected). The symbols 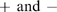 denoted the positive and negative *t* value, respectively) in two-sample two-tailed *t*-test.

For directly comparing the connectivity difference between two groups, two-sample two-tailed *t*-test was performed on all 4005 potential connections included in the 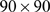 mean correlation matrices. Compared to healthy controls, 11 cross-correlations showed a statistically significant increase (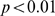, FDR corrected) in the patients group. Details can be seen in Table S3. Figure 1 shows the connectivity (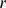) in patients was stronger than that in the controls between pairwise ROIs, e.g. lAMYG vs. lSTGp; rAMYG vs. rSTGp (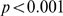, FDR corrected). 80 cross-correlations in the patients significantly decreased (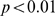, FDR corrected) compared to controls (Table S4). Furthermore, healthy controls produced significantly stronger connectivity (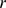) than the patients group between specific ROIs, e.g. lAMYG vs. lPCL; lPCC vs. rSFGorb; lIPG vs. rMFGorb; lIPG vs. rIPG; lIPG vs. rSPG; lPCUN vs. rPCUN; lSMG vs. rMFG; lSMG vs. rSPG; lSMG vs. rSMG; rPCC vs. rSFGorb; lIFGoper vs. rIFGoper; lIFGoper vs. rSOG; lIFGtri vs. rIFGoper; lMFGorb vs. lIPG; lSFGmed vs. rMTGp; rSFGorb vs. rTHA; rSFG vs. rTHA (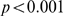, FDR corrected).

### n-to-1 Connectivity 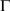


Figure S3 shows the n-to-1 total connectivity degree 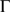 of each brain region across all subjects for each group. A larger 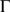 indicates that a large functional connectivity of a given region with other regions, so that the region can be considered an important node in the network [44]. The differences in n-to-1 connectivity degree between the two groups are listed in Table 1. Some ROIs showed significantly increased connectivity in mTLE, such as bilateral REG, lSFGmorb, lMTG, rIFGorb and rSFGmed (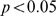, FDR corrected).

### Degree Distribution and Hubs

Details on the degree distribution, calculated as described in the Text S2, are provided in Figure S4. The nodes are connected with the largest number of other nodes in the network, i.e. the hubs, were defined as those with a degree larger than the average degree [2]. In the healthy controls, 50 nodes were found to satisfy this condition (average degree 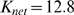), including 11 regions in the occipital cortex, 15 regions in the frontal cortex, 8 regions in the temporal cortex and 16 regions in the parietal-(pre)motor cortex. In the mTLE patients, 48 hubs were found (average degree 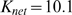), including 3 regions in the medial temporal cortex, 10 regions in the occipital cortex, 14 regions in the frontal cortex, 7 regions in the temporal cortex and 12 regions in the parietal-(pre)motor cortex, and 2 subcortical regions (for details, see Table 1 and Figure S2). For direct between-group comparisons of hubs, two-sample two-tailed *t*-test was performed on all 90 regions. Compared with the healthy controls, 5 regions (bilateral IFGoper, lPCC, lPCUN, rPreCG) showed significantly decreased values (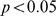, FDR corrected) in patients (Table S5).

### Altered Topological Properties of Brain Functional Network

The higher threshold resulted in a lower mean absolute clustering coefficient (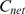) and a longer mean shortest absolute path length (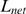) for both group. Over the whole range of 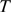 values (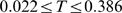) (Figure S5A) and of 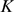 values (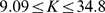) (Figure S5B), the mean absolute clustering coefficient (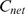) was slightly larger in controls. On the other hand, the mean shortest absolute path length, for most of the thresholds 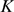, was shorter in patients compared to controls (Figure S5D).

**Figure 2:**
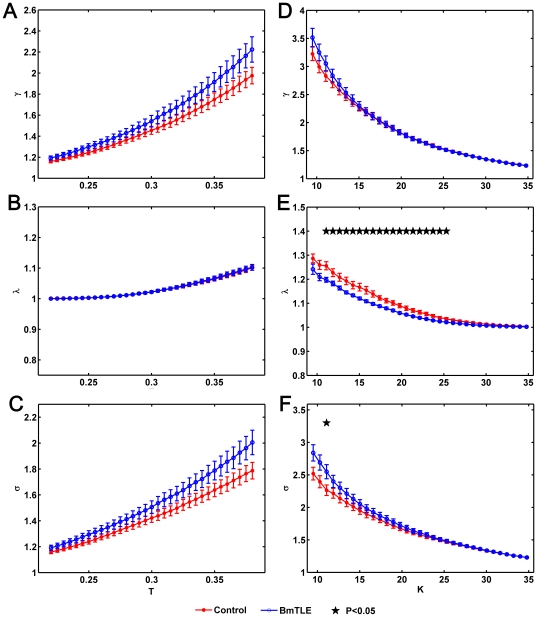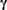, 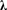 and 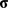 of a brain functional network. (A) 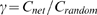 indicates the absolute clustering coefficient scaled to an equivalent parameters of a population of random graph, (B) 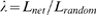 indicates the shortest absolute path length scaled to an equivalent parameters of a population of random graph and (C) 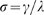 indicates the small-world-ness of network for the mTLE patients (blue circles) and healthy controls (red dots) as a function for different functional coefficient threshold 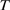 (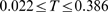). (D) 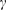, (E) 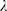 and (F) 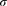 for mTLE patients (blue circles) and healthy controls (red dots) as a function of different degree of node thresholds 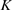 (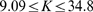). Black star markers indicate statistically significant differences between two groups (two-sample two-tailed t-test, 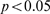, FDR corrected). Vertical bars indicate estimated standard errors.

Figure 2 shows the small-world attribute in the brain networks of both groups (see *Methods* for details about the definition of small-world attribute). It can be observed that 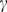 is significantly higher than 1 while 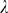 is found not to be different from 1 over the whole range of 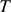 and 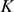 values. There are no statistically significant differences in the values of 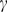, 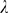 and 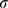 between two groups (Figure 2A–C). 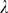 is significantly lower in the mTLE patients for most values of 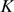 (Figure 2E). Only at a small number of connectivity values 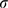 is found to be significantly increased in patients (Figure 2F).

### Relationship between Topological Measures and Clinical Variables

**Figure 3:**
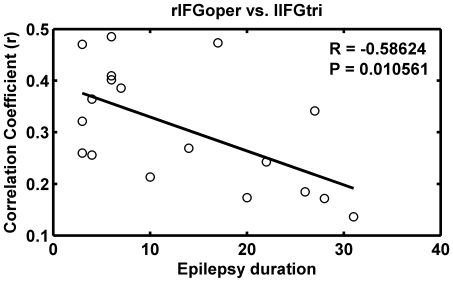
Relationship between the functional connectivity and epilepsy duration. Significant negative correlation (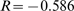, 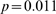) for the functional connectivity (correlation coefficient, 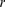) between rIFGoper and lIFGtri with the epilepsy duration.

The functional connectivity of two pairwise ROIs with significantly decreased connectivity in patients, i.e. rIFGoper vs. lIFGtri, showed a negative correlation with epilepsy duration (Figure 3). No significant correlation was found between functional connectivity and seizure frequencies.

## Discussion

By using functional connectivity and graph theoretical techniques, the present fMRI study investigated the global alterations of network properties in mTLE. The increased and decreased functional connectivity observed in specific regions might underlie the functional disruptions described in previous studies [14]–[17]. More importantly, the changes in the global topological properties including the smaller degree of connectivity, the increased n-to-1 connectivity, the smaller absolute clustering coefficients and the shorter absolute path length along with small-world properties, implicate altered whole brain network macroscopic organization [2], [3], [5], [49], [53], which extends the understanding of network mechanisms in mTLE from local characteristics to global topological properties.

### Changes in Functional Connectivity

Patients with bilateral HS were enrolled in the present study. Most of them were likely to have bilateral interictal discharges. Specific criteria was adopted to exclude a lateralization effect, despite these patients had lateralized seizure focus. A few bilateral brain regions in the mTLE patients significantly showed altered functional connectivity. Decreased connectivity was found within the frontal, parietal and occipital lobes (Figure 1 and Table S4). Notably, these areas are mostly included in the DMN [36], [54] and in the dorsal attention network, respectively [34], [35], in line with previous reports. The properties of the DMN in epilepsy patients have been documented in a few simultaneous EEG-fMRI studies, which suggested that the IEDs can suspend the normal default-mode brain function, through a pathophysiological mechanism underlying impaired consciousness in epilepsy [30]–[32], [55]. Especially, Laufs and colleagues suggested that the epileptic activity may spread from the temporal lobe into one or more functionally interconnected DMN regions [31] in TLE, and further indicated a correlation between IEDs in TLE and DMN fluctuations [31]. Our results support that the DMN is modulated in mTLE in terms of low-frequency BOLD fluctuations. IEDs are unlikely to be a result of an external requirement to perform tasks [30], and may be abnormal spontaneous neuronal events [56], [57]; hence the DMN may be momentarily suspended [30]. Although we could not directly correlate the alterations of functional connectivity to the IEDs [30], [31], [55], the interesting link between the functional connectivity and the epilepsy duration suggests that the decreased connectivity may reflect the functional impairment associated with duration of epilepsy state (Figure 3). Moreover, the decreased connectivity in the dorsal attention network confirms our previous results and suggests that the top-down attention function [58] is impaired in mTLE [16].

We also found a significantly increased functional connectivity within the medial temporal lobe, the frontal lobe, and between the parietal and frontal lobes when comparing the patients and the healthy controls (Figure 1 and Table S3). Combined with the result on decreased connectivity, the current findings demonstrate that seizures are the result of excitatory/inhibitory imbalance [15]. Furthermore, the present study could be assumed to support an alteration of the neural synchrony in temporal lobe epilepsy network [24], [59]–[61], though we have not yet solid evidence to link the BOLD fluctuations to neural oscillations [62].

Viewed as a network disorder, mTLE has been found that the widespread brain regions are functionally impaired in addition to the mesial temporal lobe. Functional connectivity MRI has been used to reveal a few of local network abnormalities in mTLE, such as the mesial temporal network [14], language network [15], attention and perceptual networks [16], [17]. Nonetheless, the current work provided new data, not only addressing the alteration in local networks, but also describing the alteration in global topological properties in mTLE patients by using a graph theoretical approach.

### Changes in Hubs

The degree distribution and hubs of healthy controls well described the properties of both resting state functional networks [5] and structural networks [6], reporting a high density of strong structural and functional core areas associated with DMN components. Buckner and colleagues have suggested that PCC/PCUN provides a key hub for overlapping connections between themselves, the medial temporal lobe, and inferior parietal lobe, which constitute the major posterior extent of the DMN [37], [63]. Because of its pivotal role, PCC/PCUN may be the first candidate to show altered properties in patients with respect to healthy controls. According to our data, not only the this area, but also other areas in the DMN, such as the IFGoper, showed a lower number of degrees in mTLE patients than healthy controls (Table 1 and Table S5). The essence of the degree that measures to which extent the node is connected to the rest of the network plays pivotal roles in the coordination of information flow [1], along with the disrupted functional connectivity of DMN discussed above, may explain why the degree of some regions in the DMN decreased more in the patients than the healthy controls. By detecting the difference of the degree of some regions between the patients and the healthy controls, we directly found the regions with the lower number of degrees, which might further confirm the dysfunction of brain network in the patients with mTLE.

### Changes in n-to-1 Connectivity 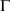


The n-to-1 connectivity 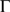 (strength of nodes) characterized how the strength of the relationship decreases with the distance between the two regions [44], [45]. The degree of a given region characterized where each connection is counted once in the unweighted connectivity matrix. On the other hand the strength of a given node is equal to the sum of exponential connection density or weight [6]. In healthy controls, the distributions of node strengths (Figure S3) show high values in the frontal cortex, temporal cortex, and partly in the parietal-(pre)motor cortex. If the functional networks are interrupted and the topological properties of the brain networks are altered in mTLE, we expect significant differences in strength values for specific brain regions between patients and healthy controls. This hypothesis was strongly supported by the statistical analysis on the strength value (Table 1). The strength values of the bilateral REG, lSFGmorb, lMTG, rIFGorb and rSFGmed significantly increased in the patients compared to controls. Understanding how the alteration of connection density of the above brain regions may yield insight into network connectivity. EEG-fMRI studies associated with epileptic discharges with DMN activity [30]–[32], [55]. However, investigations on the interruption and alteration of functional networks in the mTLE patients are currently limited. Our study hence may extend the knowledge on how the brain areas are connected in mTLE.

### Changes in Graph Theory Measures

Topological properties, including the clustering coefficient, shortest path lengths and small-world properties were altered in the mTLE patients with bitemporal damage compared to controls (Figure 2 and Figure S5). For most of the thresholds 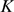, the absolute clustering coefficients (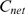) showed significantly lower values in patients, implying relatively sparse local connectedness of the brain functional networks in mTLE. This means that the local connectedness of the mTLE patients has a tendency closer to random networks, characterized by low average clustering coefficients and short mean path lengths [46]. Short absolute path lengths have been demonstrated to promote effective interactions between and across different cortical regions [1], [4], [49], [52], [53]. The shorter absolute path lengths (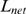) may indicate that information interactions between interconnected brain regions are faster and high efficient in mTLE. We also found that the 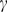 value did not show statistically significant difference between two group when the same thresholds (both for 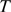 and 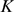) were applied, and at specific thresholds the 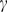 value even showed to be lower in healthy controls. More importantly, the 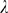 value showed a statistically significant increase for most of the thresholds 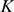, supporting the evidence that brain network of mTLE are closer to random networks. The lower absolute clustering coefficients, the shorter absolute path lengths and the small-world properties as a function of 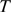 or 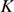 indicate that the topological measures of the brain functional networks were disrupted in mTLE. Our findings show that in the patients with mTLE, the local connectedness of the brain functional network is relatively sparse and is poorly fault tolerant in the case of loss of connectivity. Notably, it further indicates that the global topological measures of the brain functional network are disrupted in mTLE.

### Methodological Considerations and Study Limitations

Several considerations in the methodology of the current study, however, should be mentioned. Like most functional connectivity studies in brain disorders based on resting state fMRI [64], we could not eliminate the effects of physiologic noise that could be discarded using independent component analysis [65]. In the current study, in fact, we used a relatively low sampling rate (TR = 2 s) for multislice (23 slices) acquisitions. Under this sampling rate, respiratory and cardiac fluctuations may still pose a problem for fMRI time series, despite a band-pass filtering in the range 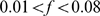 Hz is used to reduce them. These respiratory and cardiac fluctuations may reduce the specificity of low frequency fluctuations to functional connected regions [66]. Another methodology consideration is about correction for multiple comparisons. We used the Bonferroni correction when we defined the threshold 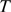 primarily in terms of the probability of the observed 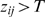 under the null hypothesis that 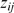 was less than an arbitrary value 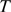. From this standpoint, we employed the strict Bonferroni correction for multiple comparisons. On the other hand, statistical comparison of functional connectivity, degree of node, n-to-1 connectivity 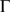, the absolute clustering coefficients 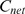, the absolute path length 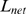, 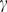, 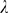 and 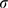 between the two groups were accomplished by two-sample two-tailed test with FDR corrected for multiple comparisons. Here, in fact, the degree of freedom (d.f.) was relatively small, and we used the relative loosen controlling (FDR) instead of the strong controlling (Bonferroni) for multiple comparisons.

This study has three main limitations. The first limitation pertains to the fact that most epilepsy imaging studies used the simultaneous EEG-fMRI technique [30]–[32], [55] to detect the activation and deactivation associated with IEDs, whereas the current study lacks of simultaneous EEG-fMRI data. It should be considered that IEDs may have occurred in the mTLE patients during resting state and could affect the finding about both functional connectivity and global topological properties. Second, although the selected patients all present bilateral HS in structural MRI and bilateral IEDs in interictal scalp-EEG, different epileptogenic lateralization among patients might still cause different alterations of functional connectivity between hemispheres. Equal number of patients with left and right mTLE was expected to avoid this bias. An additional limitation is that no sufficient special clinical variables were available for correlation with these altered topological measurements for a better understanding of the pathophysiologial mechanisms of mTLE.

### Conclusion

In conclusion, we have demonstrated that an increased functional connectivity within the medial temporal lobe, the frontal lobe, and between the parietal and frontal lobes and a decreased functional connectivity in the DMN areas in patients with mTLE. Furthermore, our results suggest that topological properties, such as the smaller degree of connectivity, the increased n-to-1 connectivity, the smaller absolute clustering coefficients and the shorter absolute path length along with small-world properties, are altered in this specific disease. We suggest that the alterations observed in functional connectivity and topological properties may be used to define tentative disease markers for mTLE.

## Supporting Information

Text S1 (0.04 MB DOC)

Text S2 (0.02 MB DOC)

Figure S1 Selection of the Upper Criteria of Small-World Regime. Largest cluster size (Giant connected cluster or largest subgraph size) as a function of T for the healthy controls (red lines) and the mTLE patients (blue lines) brain network. As expected, the percentage of the regions connected to the largest cluster decreases as a monotonically increasing function of threshold T.
(2.14 MB TIF)

Figure S2 Network Visualization of the Correlation Matrices. (A) Dorsal and lateral views of the connectivity network of healthy controls. Labels indicating anatomical regions were placed at their respective centroids. Node (individual ROIs) size was coded and colored according to their degree. Undirected edges (functional connectivity) were differently colored according to the connection strength (p≤0.001 and p≤0.0001, Bonferroni corrected) and connection polarity (positive and negative correlation coefficient r denoting the symbol ±, respectively) in the correlation matrices. (B) Dorsal and lateral views of the connectivity network of the patients.
(8.30 MB TIF)

Figure S3 n-to-1 Total Connectivity DegreeΓ. (A) Total connectivity degree for left (left column) and right (middle column) cerebral hemispheres of healthy controls. Shaded bars represent means across subjects and colored symbols indicate data for individual subjects in each group. The distribution of the total connectivity degree Γ for each group showed in the right column. Node (individual ROIs) size was coded and colored according to the total connectivity degree Γ of themselves. (B) Total connectivity degree and the distribution of the patients.
(12.19 MB TIF)

Figure S4 Degree Distribution of a Brain Functional Network. For the healthy controls (A) and the mTLE patients (B), the histogram of regional degree k_i_ distribution (Left column). Log-log plot of the cumulative probability of degree versus the degree (Right column). The blue asterisk indicates observed data, the red solid line is the best-fitting exponentially truncated power law, the dashed line is an exponential, and the dotted line is a power law.
(0.51 MB TIF)

Figure S5 C_net_ and L_net_ of a Brain Functional Network. Mean absolute clustering coefficient, C_net_, for healthy control (red dots) and mTLE patients patients (blue circles) as a function for T(0.022≤T≤0.386) (A) and as a function of K (9.09≤K≤34.8) (B). Mean shortest absolute path length, L_net_, for healthy control (red dots) and mTLE patients (blue circle) as a function for T (0.022≤T≤0.386) (C) and as a function of K (9.09≤K≤34.8) (D). Black pentagrams indicate where the statistically significant difference between two groups (two-sample two-tailed t-test,p≤0.05, FDR corrected). Vertical bars indicate estimated standard errors.
(1.99 MB TIF)

Table S1 Description of Study Patients. ED: Epilepsy duration; AO: Age onset; Fron: Frontal lobe; Temp: Temporal lobe; Par: Parietal lobe; Bi: Bilateral; L: left; R: Right; Sp: Spike; SW: Spike and wave; CPS: Complex partial seizures; SPS: Simple partial seizures; GTC: generalized tonic-clonic seizure; CBZ: carbamazepine; PHT: Phenytoin; VPA: valproate; TPM: topiramate; PB: Phenobarbital; TCHM: traditional Chinese herb medicine; CZP: clonazepam.
(0.06 MB DOC)

Table S2 Parameter Values and Goodness-of-Fit. SSE, the sum of squares due to error; R-square, the coefficient of multiple determination; Adjusted R-square, the degree of freedom adjusted R-square; RMSE, the root mean squared error; AIC, Akaike's information criterion.
(0.06 MB DOC)

Table S3 The Increased Inter-Regional Cross-Rorrelation in Patients Compared to Controls. ^a^ The regions are similar to those found in an intrinsically ‘task positive’ network, or anti-correlated with PCUN/PCC. ^b^ The regions are similar to those found in an intrinsically ‘task negative’ network, or correlated with PCUN/PCC. All p≤0.01, and asterisks (**) indicates p≤0.001, all FDR corrected.
(0.03 MB DOC)

Table S4 The Decreased Inter-Regional Cross-Correlation in Patients Compared to Controls. ^a^ The regions are similar to those found in an intrinsically ‘task positive’ network, or anti-correlated with PCUN/PCC. ^b^ The regions are similar to those found in an intrinsically ‘task negative’ network, or correlated with PCUN/PCC. All p≤0.01, and asterisks (**) indicates p≤0.001, all corrected FDR.
(0.14 MB DOC)

Table S5 Regions Showing Significantly Increased/Decreased Number of Degrees in Controls Compared to Patients. An asterisk (*) indicates p≤0.05, FDR corrected. Two-sample two-tailed t-test was performed on 90 regions. Separate columns show data for left and right cerebral hemispheres (LH and RH, respectively).
(0.09 MB DOC)
