# Supplementary material for: Altered Functional Connectivity and Small-World in Mesial Temporal Lobe Epilepsy
Source: PLoS One. 2010 Jan 8;5(1):e8525. doi: 10.1371/journal.pone.0008525 (PMC2799523; doi:10.1371/journal.pone.0008525)
Supplement: Text S1 — (0.04 MB DOC) [file pone.0008525.s001.doc]

**Text S1: Graph-theory analysis**

*Degree Distribution Fits.* Three possible forms of the degree distribution $P(k)$ [1-4] were fitted in our study: a power law [5,6], $P(k)\sim k^{-\alpha}$; an exponential [7], $P(k)\sim e^{-\alpha k}$; and an exponentially truncated power law [1,2], $P(k)\sim k^{\alpha-1}e^{k/k_{c}}$. To quantify the strength of each fit, goodness-of-fit was compared using Akaike’s information criterion for three possible forms of the degree distribution $P(k)$ [1,2].

References:

1. Bassett DS, Meyer-Lindenberg A, Achard S, Duke T, Bullmore E (2006) Adaptive reconfiguration of fractal small-world human brain functional networks. Proc Natl Acad Sci U S A 103: 19518-19523.

2. Achard S, Salvador R, Whitcher B, Suckling J, Bullmore E (2006) A resilient, low-frequency, small-world human brain functional network with highly connected association cortical hubs. J Neurosci 26: 63-72.

3. Strogatz SH (2001) Exploring complex networks. Nature 410: 268-276.

4. Palla G, Derenyi I, Farkas I, Vicsek T (2005) Uncovering the overlapping community structure of complex networks in nature and society. Nature 435: 814-818.

5. Barabasi AL, Albert R (1999) Emergence of scaling in random networks. Science 286: 509-512.

6. Eguiluz VM, Chialvo DR, Cecchi GA, Baliki M, Apkarian AV (2005) Scale-free brain functional networks. Phys Rev Lett 94: 018102.

7. Hagmann P, Cammoun L, Gigandet X, Meuli R, Honey CJ, et al. (2008) Mapping the structural core of human cerebral cortex. PLoS Biol 6: e159.
