# Supplementary material for: Altered Functional Connectivity and Small-World in Mesial Temporal Lobe Epilepsy
Source: PLoS One. 2010 Jan 8;5(1):e8525. doi: 10.1371/journal.pone.0008525 (PMC2799523; doi:10.1371/journal.pone.0008525)
Supplement: Text S2 — (0.02 MB DOC) [file pone.0008525.s002.doc]

**Text S2: Degree distribution**

There was further evidence for scale-invariant topology when we compared the degree distributions of the scale-specific networks in each group (Figure S4). For each group, the degree distribution was fitted by three possible forms of the degree distribution $P(k)$ (see *Methods and Methods*)*. Am*ong these, the exponentially truncated power law was the best-fitting model for the degree distribution (Figure S4) rather than the scale-free for each group (Table S1 for parameter values and goodness-of-fit).
