# Supplementary figures and images for: Altered Functional Connectivity and Small-World in Mesial Temporal Lobe Epilepsy
Source: PLoS One. 2010 Jan 8;5(1):e8525. doi: 10.1371/journal.pone.0008525 (PMC2799523; doi:10.1371/journal.pone.0008525)

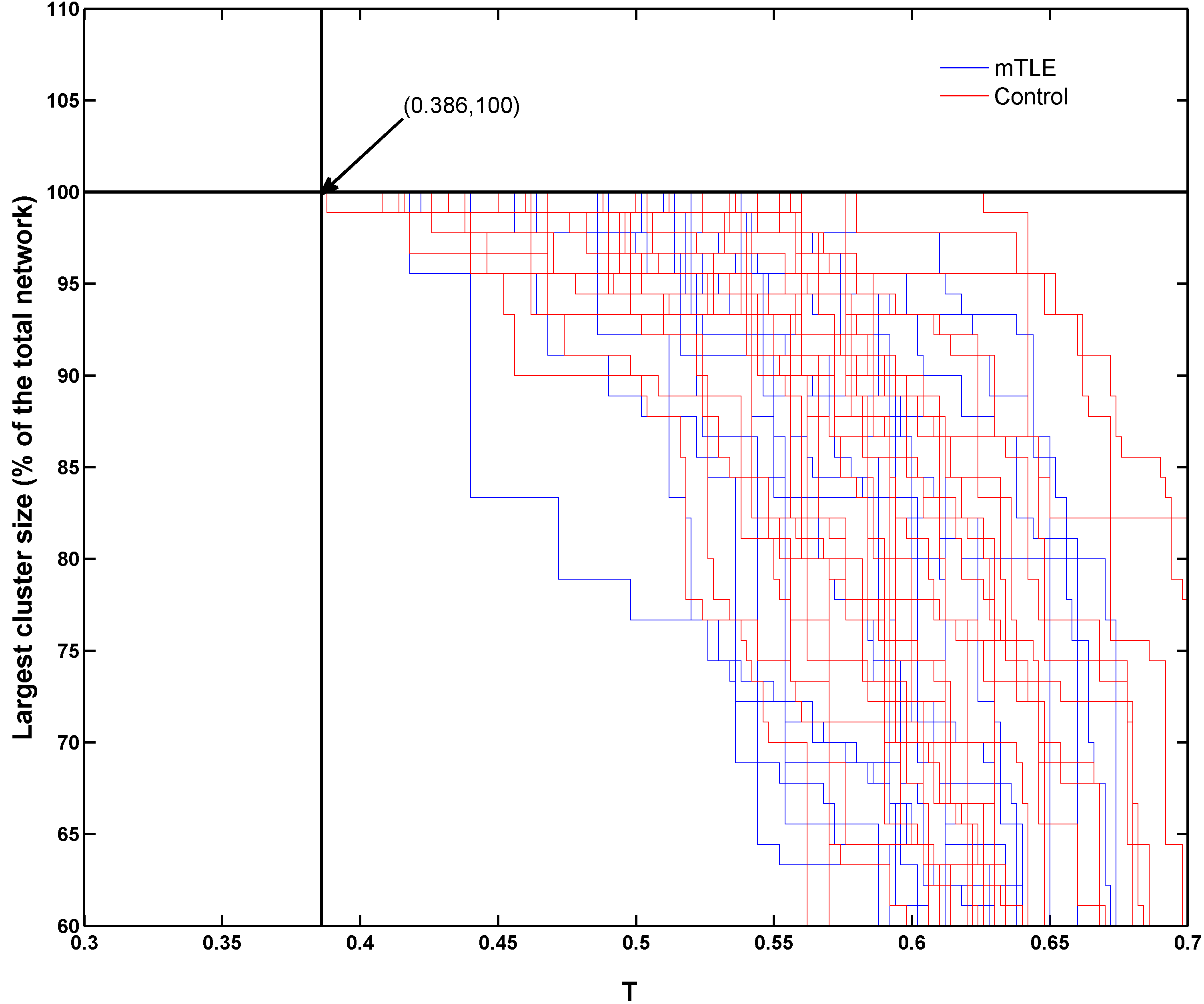

Supplement: Figure S1 — Selection of the Upper Criteria of Small-World Regime. Largest cluster size (Giant connected cluster or largest subgraph size) as a function of T for the healthy controls (red lines) and the mTLE patients (blue lines) brain network. As expected, the percentage of the regions connected to the largest cluster decreases as a monotonically increasing function of threshold T. (2.14 MB TIF) [file pone.0008525.s003.tif]

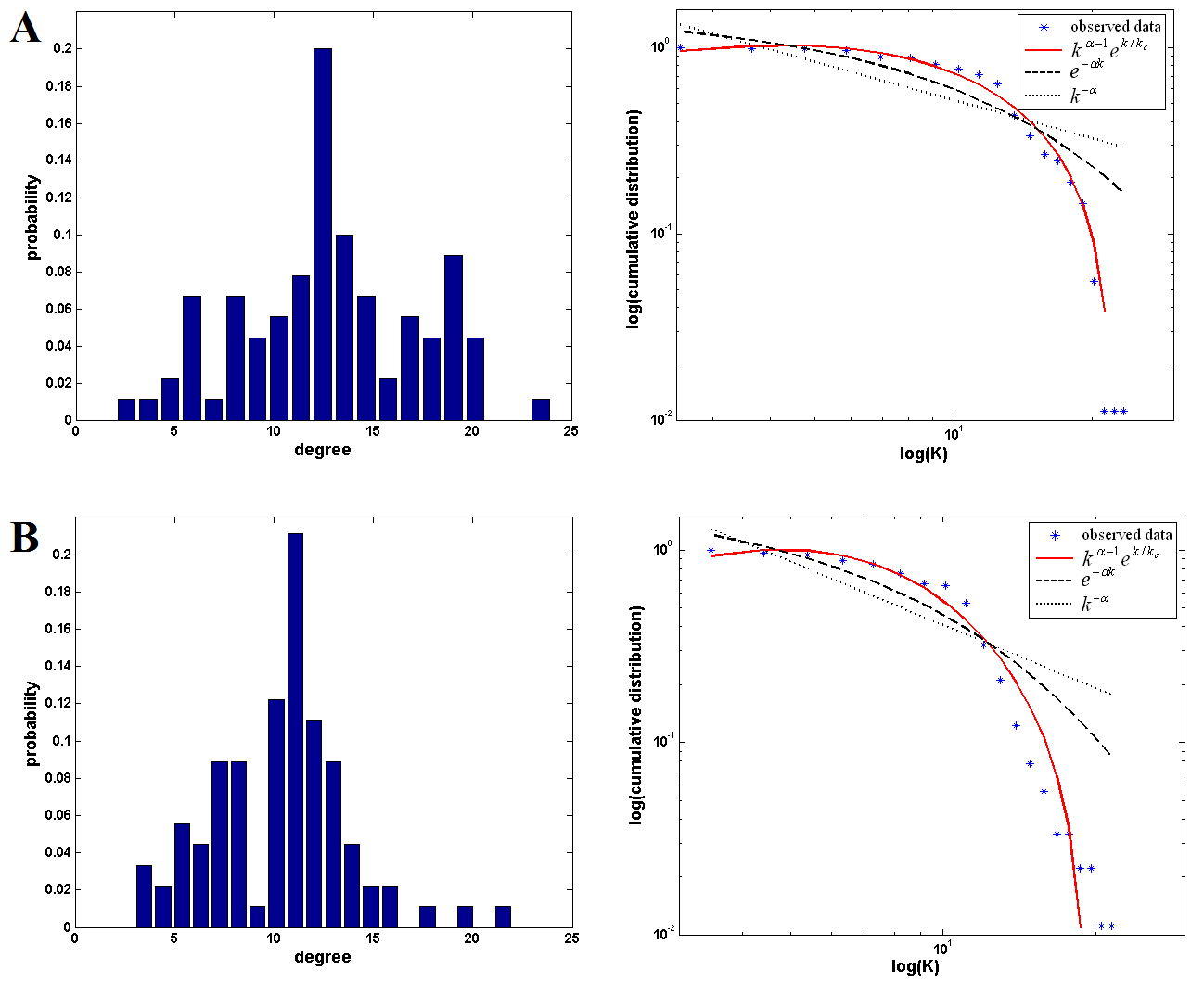

Supplement: Figure S4 — Degree Distribution of a Brain Functional Network. For the healthy controls (A) and the mTLE patients (B), the histogram of regional degree ki distribution (Left column). Log-log plot of the cumulative probability of degree versus the degree (Right column). The blue asterisk indicates observed data, the red solid line is the best-fitting exponentially truncated power law, the dashed line is an exponential, and the dotted line is a power law. (0.51 MB TIF) [file pone.0008525.s006.tif]

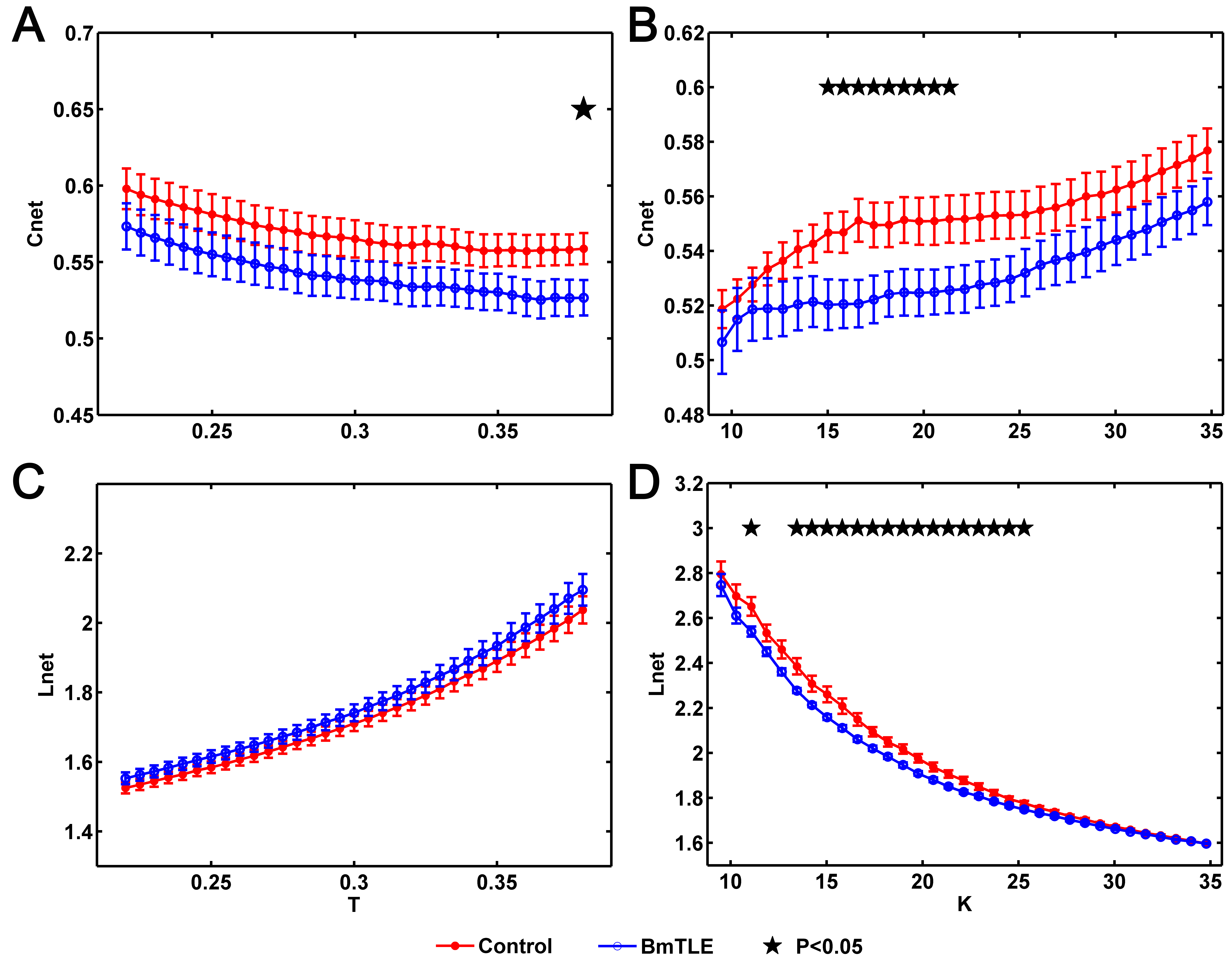

Supplement: Figure S5 — Cnet and Lnet of a Brain Functional Network. Mean absolute clustering coefficient, Cnet, for healthy control (red dots) and mTLE patients patients (blue circles) as a function for T(0.022≤T≤0.386) (A) and as a function of K (9.09≤K≤34.8) (B). Mean shortest absolute path length, Lnet, for healthy control (red dots) and mTLE patients (blue circle) as a function for T (0.022≤T≤0.386) (C) and as a function of K (9.09≤K≤34.8) (D). Black pentagrams indicate where the statistically significant difference between two groups (two-sample two-tailed t-test,p≤0.05, FDR corrected). Vertical bars indicate estimated standard errors. (1.99 MB TIF) [file pone.0008525.s007.tif]
