# Supplementary material for: Altered Functional Connectivity and Small-World in Mesial Temporal Lobe Epilepsy
Source: PLoS One. 2010 Jan 8;5(1):e8525. doi: 10.1371/journal.pone.0008525 (PMC2799523; doi:10.1371/journal.pone.0008525)
Supplement: Table S1 — Description of Study Patients. ED: Epilepsy duration; AO: Age onset; Fron: Frontal lobe; Temp: Temporal lobe; Par: Parietal lobe; Bi: Bilateral; L: left; R: Right; Sp: Spike; SW: Spike and wave; CPS: Complex partial seizures; SPS: Simple partial seizures; GTC: generalized tonic-clonic seizure; CBZ: carbamazepine; PHT: Phenytoin; VPA: valproate; TPM: topiramate; PB: Phenobarbital; TCHM: traditional Chinese herb medicine; CZP: clonazepam. (0.06 MB DOC) [file pone.0008525.s008.doc]

**Table S1.** Description of Study Patients

| Patient # | Sex | Age | ED | AO | Interictal EEG | Structural MRI | Seizure types | Therapy |
| --- | --- | --- | --- | --- | --- | --- | --- | --- |
| 1 | F | 17 | 3 | 14 | Bi Temp/Fron SW, Sp | Bi-HS | CPS/GTC | VPA |
| 2 | F | 27 | 26 | 1 | Bi Temp/Fron Sp | Bi-HS | SPS/CPS/GTC | VPA/PHT |
| 3 | M | 17 | 4 | 13 | L Temp/central Sp, R Temp Sp | Bi-HS | GTC | VPA/TPM |
| 4 | M | 32 | 28 | 4 | Bi Temp Sp | Bi-HS | SPS/CPS/GTC | VPA/PHT/TCHM |
| 5 | M | 23 | 6 | 17 | L Temp/central Sp, Widespread SW | Bi-HS | SPS/CPS/GTC | VPA/TPM/TCHM |
| 6 | M | 18 | 6 | 12 | Bi Temp/Fron slow wave, L Temp Sp | Bi-HS | SPS/GTC | VPA/CZP |
| 7 | M | 19 | 3 | 16 | Bi Fron/Temp/Par SW | Bi-HS | SPS/CPS/GTC | VPA |
| 8 | M | 20 | 3 | 17 | Bi Temp Sp | Bi-HS | SPS/GTC | VPA |
| 9 | M | 17 | 6 | 11 | Bi Temp/central Sp | Bi-HS | GTC | VPA |
| 10 | M | 27 | 27 | 1 | Bi Temp/Fron Sp | Bi-HS | SPS/CPS/GTC | CBZ/CZP |
| 11 | F | 19 | 14 | 5 | Bi Temp Sp, Front SW | Bi-HS | SPS | PB/TCHM |
| 12 | M | 24 | 22 | 2 | Bi Fron/Temp/central Sp | Bi-HS | CPS/GTC | VPA |
| 13 | F | 28 | 10 | 18 | Bi Front SW | Bi-HS | CPS/GTC | PHT/VPA |
| 14 | M | 23 | 7 | 16 | Bi Temp/Fron SW | Bi-HS | CPS | CBZ/PHT |
| 15 | F | 32 | 31 | 1 | Bi Temp/Par Sp | Bi-HS | SPS/CPS/GTC | VPA/CBZ |
| 16 | F | 19 | 4 | 15 | L Temp/Fron Sp, Widespread SW | Bi-HS | GTC | No |
| 17 | M | 17 | 17 | 1 | L Temp/Fron Sp ,Widespread SW | Bi-HS | CPS/GTC | CBZ/VPA |
| 18 | F | 51 | 20 | 31 | Widespread SW | Bi-HS | SPS/CPS/GTC | PB/PHT |

ED: Epilepsy duration; AO: Age onset; Fron: Frontal lobe; Temp: Temporal lobe; Par: Parietal lobe; Bi: Bilateral; L: left; R: Right; Sp: Spike; SW: Spike and wave; CPS: Complex partial seizures; SPS: Simple partial seizures; GTC: generalized tonic-clonic seizure; CBZ: carbamazepine; PHT: Phenytoin; VPA: valproate; TPM: topiramate; PB: Phenobarbital; TCHM: traditional Chinese herb medicine; CZP: clonazepam.
