# Supplementary material for: Altered Functional Connectivity and Small-World in Mesial Temporal Lobe Epilepsy
Source: PLoS One. 2010 Jan 8;5(1):e8525. doi: 10.1371/journal.pone.0008525 (PMC2799523; doi:10.1371/journal.pone.0008525)
Supplement: Table S2 — Parameter Values and Goodness-of-Fit. SSE, the sum of squares due to error; R-square, the coefficient of multiple determination; Adjusted R-square, the degree of freedom adjusted R-square; RMSE, the root mean squared error; AIC, Akaike's information criterion. (0.06 MB DOC) [file pone.0008525.s009.doc]

**Table S2.** Parameter Values and Goodness-of-Fit

| Degree distribution | Controls | mTLE |
| --- | --- | --- |
| $P(k)\sim k^{-\alpha}$ | | |
| $\alpha$ | 0.6817$\pm$0.26 | 1.088$\pm$0.36 |
| SSE | 0.9238 | 0.7303 |
| R-square | 0.6621 | 0.7474 |
| Adjusted R-square | 0.6433 | 0.7334 |
| RMSE | 0.2265 | 0.2014 |
| AIC | -57.49 | -62.20 |
| $P(k)\sim e^{-\alpha k}$ | | |
| $\alpha$ | 0.0960$\pm$0.02 | 0.1476$\pm$0.04 |
| SSE | 0.3695 | 0.3216 |
| R-square | 0.8649 | 0.8888 |
| Adjusted R-square | 0.8574 | 0.8826 |
| RMSE | 0.1433 | 0.1337 |
| AIC | -75.83 | -94.18 |
| $P(k)\sim k^{\alpha-1}e^{k/k_{c}}$ | | |
| $\alpha$ | 1.4370$\pm$0.34 | 2.5600$\pm$0.83 |
| $k_{c}$ | 3.7598$\pm$0.85 | 3.0618$\pm$0.86 |
| SSE | 0.1025 | 0.0796 |
| R-square | 0.9625 | 0.9725 |
| Adjusted R-square | 0.9581 | 0.9692 |
| RMSE | 0.0777 | 0.0684 |
| AIC | -97.47 | -102.53 |

SSE, the sum of squares due to error; R-square, the coefficient of multiple determination; Adjusted R-square, the degree of freedom adjusted R-square; RMSE, the root mean squared error; AIC, Akaike’s information criterion.
