# Supplementary material for: Altered Functional Connectivity and Small-World in Mesial Temporal Lobe Epilepsy
Source: PLoS One. 2010 Jan 8;5(1):e8525. doi: 10.1371/journal.pone.0008525 (PMC2799523; doi:10.1371/journal.pone.0008525)
Supplement: Table S3 — The Increased Inter-Regional Cross-Rorrelation in Patients Compared to Controls. a The regions are similar to those found in an intrinsically ‘task positive’ network, or anti-correlated with PCUN/PCC. b The regions are similar to those found in an intrinsically ‘task negative’ network, or correlated with PCUN/PCC. All p≤0.01, and asterisks (**) indicates p≤0.001, all FDR corrected. (0.03 MB DOC) [file pone.0008525.s010.doc]

**Table S3. The Increased Inter-Regional Cross-Rorrelation in Patients Compared to Controls**

| Region 1 | Classification | Region 2 | Classification | P value | T value |
| --- | --- | --- | --- | --- | --- |
| lAMYG | Medial Temporal | lSTGp | Medial Temporal | 0.0007 | 3.6412** |
| rAMYG | Medial Temporal | rSTGp | Medial Temporal | 0.0004 | 3.8103** |
| lAMYG | Medial Temporal | lMTGp | Medial Temporal | 0.0085 | 2.7564 |
| lHIP ^b^ | Medial Temporal | lMTGp | Medial Temporal | 0.0072 | 2.8207 |
| lIFGorb | Frontal | rPCC ^b^ | Parietal-(pre)Motor | 0.0067 | 2.8483 |
| lIFGorb | Frontal | rREG | Frontal | 0.0073 | 2.8168 |
| lSFGmorb | Frontal | rIFGoper ^a^ | Frontal | 0.0070 | 2.8495 |
| lSFGmed | Frontal | rMFG ^a^ | Frontal | 0.0077 | 2.7955 |
| lREG | Frontal | rPoCG | Parietal-(pre)Motor | 0.0043 | 3.0163 |
| lIOG ^a^ | Occipital | rSFGmed | Frontal | 0.0030 | 3.1442 |
| lPCL | Parietal-(pre)Motor | rSFGmed | Frontal | 0.0078 | 2.7906 |

^a^The regions are similar to those found in an intrinsically ‘‘task positive’’ network, or anti-correlated with PCUN/PCC.

^b^The regions are similar to those found in an intrinsically ‘‘task negative’’ network, or correlated with PCUN/PCC.

All $p<0.01$, and asterisks (**) indicates $p<0.001$, all FDR corrected.
