# Supplementary material for: Altered Functional Connectivity and Small-World in Mesial Temporal Lobe Epilepsy
Source: PLoS One. 2010 Jan 8;5(1):e8525. doi: 10.1371/journal.pone.0008525 (PMC2799523; doi:10.1371/journal.pone.0008525)
Supplement: Table S4 — The Decreased Inter-Regional Cross-Correlation in Patients Compared to Controls. a The regions are similar to those found in an intrinsically ‘task positive’ network, or anti-correlated with PCUN/PCC. b The regions are similar to those found in an intrinsically ‘task negative’ network, or correlated with PCUN/PCC. All p≤0.01, and asterisks (**) indicates p≤0.001, all corrected FDR. (0.14 MB DOC) [file pone.0008525.s011.doc]

**Table S4. The Decreased Inter-Regional Cross-Correlation in Patients Compared to Controls**

| Region 1 | Classification | Region 2 | Classification | P value | T value |
| --- | --- | --- | --- | --- | --- |
| lAMYG | Medial Temporal | lPCL | Parietal-(pre)Motor | 0.0000 | -4.6997** |
| lPCC ^b^ | Parietal-(pre)Motor | rSFGorb | Frontal | 0.0001 | -4.1727** |
| lIPG ^b^ | Parietal-(pre)Motor | rMFGorb | Frontal | 0.0000 | -4.5852** |
| lIPG ^b^ | Parietal-(pre)Motor | rIPG ^a^ | Parietal-(pre)Motor | 0.0001 | -4.1931** |
| lIPG ^b^ | Parietal-(pre)Motor | rSPG ^a^ | Parietal-(pre)Motor | 0.0006 | -3.6967** |
| lPCUN ^b^ | Parietal-(pre)Motor | rPCUN ^b^ | Parietal-(pre)Motor | 0.0008 | -3.5814** |
| lSMG | Parietal-(pre)Motor | rMFG ^a^ | Frontal | 0.0003 | -3.8476** |
| lSMG | Parietal-(pre)Motor | rSPG ^a^ | Parietal-(pre)Motor | 0.0006 | -3.7083** |
| lSMG | Parietal-(pre)Motor | rSMG | Parietal-(pre)Motor | 0.0002 | -4.0973** |
| rPCC ^b^ | Parietal-(pre)Motor | rSFGorb | Frontal | 0.0007 | -3.6652** |
| lIFGoper ^a^ | Frontal | rIFGoper ^a^ | Frontal | 0.0004 | -3.8681** |
| lIFGoper ^a^ | Frontal | rSOG | Occipital | 0.0004 | -3.8127** |
| lIFGtri | Frontal | rIFGoper ^a^ | Frontal | 0.0004 | -3.8387** |
| lMFGorb | Frontal | lIPG ^b^ | Parietal-(pre)Motor | 0.0002 | -4.1430** |
| lSFGmed | Frontal | rMTGp | Medial Temporal | 0.0005 | -3.7800** |
| rSFGorb | Frontal | rTHA | Subcortical | 0.0008 | -3.6007** |
| rSFG ^b^ | Frontal | rTHA | Subcortical | 0.0008 | -3.5945** |
| lANG ^b^ | Parietal-(pre)Motor | lSFGmed | Frontal | 0.0051 | -2.9514 |
| lANG ^b^ | Parietal-(pre)Motor | rSFGmed | Frontal | 0.0021 | -3.2643 |
| lANG ^b^ | Parietal-(pre)Motor | rSFG ^b^ | Frontal | 0.0046 | -2.9911 |
| lMCC | Parietal-(pre)Motor | rTHA | Subcortical | 0.0075 | -2.8059 |
| lPCC ^b^ | Parietal-(pre)Motor | rSFG ^b^ | Frontal | 0.0062 | -2.8810 |
| lPCL | Parietal-(pre)Motor | rAMYG | Medial Temporal | 0.0022 | -3.2635 |
| lIPG ^b^ | Parietal-(pre)Motor | rMFG ^a^ | Frontal | 0.0009 | -3.5599 |
| lIPG ^b^ | Parietal-(pre)Motor | rSFGorb | Frontal | 0.0026 | -3.1908 |
| lSPG ^a^ | Parietal-(pre)Motor | rMFGorb | Frontal | 0.0010 | -3.5476 |
| lPreCG ^a^ | Parietal-(pre)Motor | rHIP ^b^ | Medial Temporal | 0.0087 | -2.7491 |
| lPreCG ^a^ | Parietal-(pre)Motor | rSOG | Occipital | 0.0058 | -2.9032 |
| lPreCG ^a^ | Parietal-(pre)Motor | rSPG ^a^ | Parietal-(pre)Motor | 0.0060 | -2.8889 |
| lSMA ^a^ | Parietal-(pre)Motor | rTHA | Subcortical | 0.0041 | -3.0336 |
| lSMG | Parietal-(pre)Motor | rMFGorb | Frontal | 0.0069 | -2.8394 |
| rANG ^b^ | Parietal-(pre)Motor | rSFGorb | Frontal | 0.0033 | -3.1100 |
| rMCC | Parietal-(pre)Motor | rSFG ^b^ | Frontal | 0.0074 | -2.8116 |
| rMCC | Parietal-(pre)Motor | rTHA | Subcortical | 0.0047 | -2.9786 |
| rPCC ^b^ | Parietal-(pre)Motor | rSFG ^b^ | Frontal | 0.0057 | -2.9087 |
| lACC ^b^ | Frontal | lOLF | Subcortical | 0.0044 | -3.0044 |
| lIFGoper ^a^ | Frontal | lIOG ^a^ | Occipital | 0.0070 | -2.8316 |
| lIFGoper ^a^ | Frontal | lMOG | Occipital | 0.0029 | -3.1563 |
| lIFGoper ^a^ | Frontal | rMFG ^a^ | Frontal | 0.0041 | -3.0329 |
| lIFGoper ^a^ | Frontal | rMOG | Occipital | 0.0032 | -3.1192 |
| lIFGoper ^a^ | Frontal | rSPG ^a^ | Parietal-(pre)Motor | 0.0034 | -3.1009 |
| lIFGtri | Frontal | lMFGorb | Frontal | 0.0062 | -2.8770 |
| lIFGtri | Frontal | lMFG ^a^ | Frontal | 0.0038 | -3.0581 |
| lIFGtri | Frontal | rMFGorb | Frontal | 0.0009 | -3.5574 |
| lIFGtri | Frontal | rMFG ^a^ | Frontal | 0.0010 | -3.5304 |
| lIFGtri | Frontal | rSPG ^a^ | Parietal-(pre)Motor | 0.0017 | -3.3542 |
| lSFGmorb | Frontal | rMTGp | Medial Temporal | 0.0027 | -3.1814 |
| lMFGorb | Frontal | lFG ^a^ | Occipital | 0.0025 | -3.2164 |
| lMFGorb | Frontal | lITG ^b^ | Temporal | 0.0039 | -3.0480 |
| lMFG ^a^ | Frontal | rANG ^b^ | Parietal-(pre)Motor | 0.0049 | -2.9699 |
| lSFGmed | Frontal | rSFGmed | Frontal | 0.0056 | -2.9181 |
| lSFGorb | Frontal | rCAL | Occipital | 0.0046 | -2.9903 |
| lSFGorb | Frontal | rCUN | Occipital | 0.0073 | -2.8186 |
| lSFG ^b^ | Frontal | rCAL | Occipital | 0.0086 | -2.7538 |
| rSFGmorb | Frontal | rMTGp | Medial Temporal | 0.0030 | -3.1431 |
| rMFGorb | Frontal | rTHA | Subcortical | 0.0069 | -2.8392 |
| rMFG ^a^ | Frontal | rSPG ^a^ | Parietal-(pre)Motor | 0.0035 | -3.0953 |
| rSFGmed | Frontal | rMTGp | Medial Temporal | 0.0012 | -3.4793 |
| rSFGorb | Frontal | rHIP ^b^ | Medial Temporal | 0.0020 | -3.2860 |
| rSFG ^b^ | Frontal | rMTG ^a^ | Temporal | 0.0056 | -2.9137 |
| lHES | Temporal | lSTG | Temporal | 0.0077 | -2.8080 |
| lINS ^a^ | Temporal | lROL | Parietal-(pre)Motor | 0.0060 | -2.8884 |
| lINS ^a^ | Temporal | lSMG | Parietal-(pre)Motor | 0.0049 | -2.9597 |
| lINS ^a^ | Temporal | lSTG | Temporal | 0.0062 | -2.8814 |
| lROL | Parietal-(pre)Motor | rSPG ^a^ | Parietal-(pre)Motor | 0.0038 | -3.0590 |
| lROL | Parietal-(pre)Motor | rPoCG | Parietal-(pre)Motor | 0.0043 | -3.0139 |
| lROL | Parietal-(pre)Motor | rSMG | Parietal-(pre)Motor | 0.0010 | -3.5314 |
| lSTG | Temporal | rSMG | Parietal-(pre)Motor | 0.0014 | -3.4198 |
| lINS ^a^ | Temporal | rROL | Parietal-(pre)Motor | 0.0046 | -2.9932 |
| lLING | Occipital | rROL | Parietal-(pre)Motor | 0.0025 | -3.2106 |
| lMOG | Occipital | rHIP ^b^ | Medial Temporal | 0.0037 | -3.0692 |
| rCAL | Occipital | rITG ^b^ | Temporal | 0.0038 | -3.0571 |
| lSOG | Occipital | rHIP ^b^ | Medial Temporal | 0.0068 | -2.8448 |
| lTHA | Subcortical | rSFGorb | Frontal | 0.0053 | -2.9372 |
| lHIP ^b^ | Medial Temporal | lSOG | Occipital | 0.0056 | -2.9168 |
| rHIP ^b^ | Medial Temporal | rIOG ^a^ | Occipital | 0.0019 | -3.3100 |
| rHIP ^b^ | Medial Temporal | rMOG | Occipital | 0.0046 | -2.9889 |
| rHIP ^b^ | Medial Temporal | rSOG | Occipital | 0.0009 | -3.5660 |
| rHIP ^b^ | Medial Temporal | rPoCG | Parietal-(pre)Motor | 0.0076 | -2.8024 |
| rHIP ^b^ | Medial Temporal | rPreCG ^a^ | Parietal-(pre)Motor | 0.0009 | -3.5583 |

^a^The regions are similar to those found in an intrinsically ‘‘task positive’’ network, or anti-correlated with PCUN/PCC.

^b^The regions are similar to those found in an intrinsically ‘‘task negative’’ network, or correlated with PCUN/PCC.

All $p<0.01$, and asterisks (**) indicates $p<0.001$, all corrected FDR.
