# Supplementary material for: Altered Functional Connectivity and Small-World in Mesial Temporal Lobe Epilepsy
Source: PLoS One. 2010 Jan 8;5(1):e8525. doi: 10.1371/journal.pone.0008525 (PMC2799523; doi:10.1371/journal.pone.0008525)
Supplement: Table S5 — Regions Showing Significantly Increased/Decreased Number of Degrees in Controls Compared to Patients. An asterisk (*) indicates p≤0.05, FDR corrected. Two-sample two-tailed t-test was performed on 90 regions. Separate columns show data for left and right cerebral hemispheres (LH and RH, respectively). (0.09 MB DOC) [file pone.0008525.s012.doc]

**Table S5. Regions Showing Significantly Increased/Decreased Number of Degrees in Controls Compared to Patients**

|  |  | T value | | P value | |
| --- | --- | --- | --- | --- | --- |
|  |  | mTLE vs. Controls | | mTLE vs. Controls | |
| Region name | Abbreviation | LH | RH | LH | RH |
| *Medial Temporal* |  |  |  |  |  |
| Amygdala | AMYG | 0.64693 | -0.20892 | 0.52111 | 0.83550 |
| Hippocampus | HIP | 0.66093 | -0.88682 | 0.51218 | 0.38011 |
| Parahippocampal gyrus | PHIP | 0.84292 | 0.39561 | 0.40394 | 0.69435 |
| Middle temporal gyrus, temporal pole | MTGp | -0.81642 | -1.40911 | 0.41876 | 0.16599 |
| Superior temporal gyrus, temporal pole | STGp | -1.16799 | -0.47987 | 0.24925 | 0.63375 |
| *Subcortical* |  |  |  |  |  |
| Caudate nucleus | CAU | -0.13341 | -0.19198 | 0.89449 | 0.84866 |
| Olfactory cortex | OLF | -0.54190 | -0.99983 | 0.59069 | 0.32299 |
| Pallidum | PAL | -0.16025 | -0.18492 | 0.87343 | 0.85416 |
| Putamen | PUT | 0.24373 | 0.00966 | 0.80860 | 0.99234 |
| Thalamus | THA | 0.08150 | -0.25034 | 0.93542 | 0.80352 |
| *Occipital* |  |  |  |  |  |
| Calcarine fissure | CAL | -0.69306 | -1.13862 | 0.49200 | 0.26117 |
| Cuneus | CUN | -1.48044 | -1.20470 | 0.14605 | 0.23491 |
| Fusiform gyrus | FG | -0.98189 | -1.81619 | 0.33165 | 0.07632 |
| Lingual gyrus | LING | -1.25623 | -0.76368 | 0.21581 | 0.44923 |
| Inferior occipital gyrus | IOG | -0.28973 | -0.17239 | 0.77342 | 0.86394 |
| Middle occipital gyrus | MOG | -1.59392 | -1.51600 | 0.11828 | 0.13684 |
| Superior occipital gyrus | SOG | -0.40039 | -1.38175 | 0.69086 | 0.17418 |
| *Frontal* |  |  |  |  |  |
| Anterior cingulate gyrus | ACC | -0.75916 | -0.84620 | 0.45190 | 0.40213 |
| Inferior frontal gyrus, opercular | IFGoper | -2.13156 | -2.26047 | **0.03880*** | **0.02891*** |
| Inferior frontal gyrus, orbital | IFGorb | -0.62014 | 0.25468 | 0.53844 | 0.80018 |
| Inferior frontal gyrus, triangular | IFGtri | -1.77204 | -1.06879 | 0.08347 | 0.29113 |
| Superior frontal gyrus, medial orbital | SFGmorb | -0.70591 | -1.17310 | 0.48405 | 0.24721 |
| Middle frontal gyrus, orbital | MFGorb | -1.45298 | -0.87705 | 0.15349 | 0.38533 |
| Middle frontal gyrus | MFG | -1.49943 | -1.32487 | 0.14107 | 0.19221 |
| Superior frontal gyrus, medial | SFGmed | 0.17116 | 0.00695 | 0.86490 | 0.99449 |
| Superior frontal gyrus, orbital | SFGorb | 0.99137 | -0.55470 | 0.32705 | 0.58198 |
| Superior frontal gyrus | SFG | -0.56738 | -1.71931 | 0.57341 | 0.09275 |
| Gyrus rectus | REG | -0.82254 | -0.18615 | 0.41531 | 0.85320 |
| *Temporal* |  |  |  |  |  |
| Heschl gyrus | HES | -1.00318 | -0.31603 | 0.32139 | 0.75351 |
| Insula | INS | -1.08975 | -1.55311 | 0.28189 | 0.12773 |
| Inferior temporal gyrus | ITG | -1.67941 | -1.48969 | 0.10032 | 0.14361 |
| Middle temporal gyrus | MTG | 0.59380 | -1.59966 | 0.55576 | 0.11700 |
| Superior temporal gyrus | STG | -0.20120 | -0.27550 | 0.84149 | 0.78425 |
| *Parietal-(pre)Motor* |  |  |  |  |  |
| Rolandic operculum | ROL | -1.37190 | -2.00452 | 0.17721 | 0.05134 |
| Angular gyrus | ANG | -1.23942 | -1.46648 | 0.22191 | 0.14979 |
| Median cingulate gyrus | MCC | -1.89368 | -1.66706 | 0.06501 | 0.10277 |
| Posterior cingulate gyrus | PCC | -2.39403 | -2.00322 | **0.02110*** | 0.05148 |
| Paracentral lobule | PCL | 0.49201 | 0.36294 | 0.62521 | 0.71843 |
| Inferior parietal gyrus | IPG | -1.42908 | -1.30513 | 0.16021 | 0.19879 |
| Superior parietal gyrus | SPG | -1.58868 | -1.68117 | 0.11946 | 0.09998 |
| Postcentral gyrus | PoCG | -0.48636 | -1.20456 | 0.62918 | 0.23496 |
| Precentral gyrus | PreCG | -1.46371 | -2.15770 | 0.15055 | **0.03658*** |
| Precuneus | PCUN | -2.15793 | -1.26760 | **0.03656*** | 0.21176 |
| Supplementary motor area | SMA | -0.94495 | 0.12217 | 0.34997 | 0.90333 |
| Supramarginal gyrus | SMG | -1.99725 | -1.82600 | 0.05215 | 0.07480 |

An asterisk (*) indicates $p<0.05$, FDR corrected. Two-sample two-tailed *t-*test was performed on 90 regions. Separate columns show data for left and right cerebral hemispheres (LH and RH, respectively).
